# OccuPeak: ChIP-Seq Peak Calling Based on Internal Background Modelling

**DOI:** 10.1371/journal.pone.0099844

**Published:** 2014-06-17

**Authors:** Bouke A. de Boer, Karel van Duijvenboden, Malou van den Boogaard, Vincent M. Christoffels, Phil Barnett, Jan M. Ruijter

**Affiliations:** Department of Anatomy, Embryology & Physiology, Academic Medical Centre, Amsterdam, The Netherlands; University of Cologne, Germany

## Abstract

ChIP-seq has become a major tool for the genome-wide identification of transcription factor binding or histone modification sites. Most peak-calling algorithms require input control datasets to model the occurrence of background reads to account for local sequencing and GC bias. However, the GC-content of reads in Input-seq datasets deviates significantly from that in ChIP-seq datasets. Moreover, we observed that a commonly used peak calling program performed equally well when the use of a simulated uniform background set was compared to an Input-seq dataset. This contradicts the assumption that input control datasets are necessary to fatefully reflect the background read distribution. Because the GC-content of the abundant single reads in ChIP-seq datasets is similar to those of randomly sampled regions we designed a peak-calling algorithm with a background model based on overlapping single reads. The application, OccuPeak, uses the abundant low frequency tags present in each ChIP-seq dataset to model the background, thereby avoiding the need for additional datasets. Analysis of the performance of OccuPeak showed robust model parameters. Its measure of peak significance, the excess ratio, is only dependent on the tag density of a peak and the global noise levels. Compared to the commonly used peak-calling applications MACS and CisGenome, OccuPeak had the highest sensitivity in an enhancer identification benchmark test, and performed similar in an overlap tests of transcription factor occupation with DNase I hypersensitive sites and H3K27ac sites. Moreover, peaks called by OccuPeak were significantly enriched with cardiac disease-associated SNPs. OccuPeak runs as a standalone application and does not require extensive tweaking of parameters, making its use straightforward and user friendly. Availability: http://occupeak.hfrc.nl

## Glossary

**Read**: Sequenced DNA fragment

**Dataset**: List of reads originating from a sequence run

**ChIP-seq dataset**: Dataset resulting from a ChIP-seq experiment after immunoprecipitation with a specific antibody

**Input-seq dataset**: Dataset resulting from a sequencing experiment without immunoprecipitation or precipitation without specific antibody

**Tag**: Read aligned to the genome

**Region**: Part of the genome covered by overlapping tags

**Peak**: Region covered by a number of tags that exceeds the threshold of the applied peak-calling algorithm

**Noise** or **Background**: Region covered by a number of tags which does not exceed the threshold of the applied peak-calling algorithm

**Excess Ratio** **(ER)**: Ratio of the observed number of regions and the expected number of regions with *n or more* tags. The expected number is calculated from the proposed model for the distribution of background tags over the chromosome

**Sensitivity**: fraction of the actual peaks that is correctly called as peak ("true positive peaks").

**Specificity** is statistically defined as "the fraction of true negatives". Because the population of negatives cannot be properly defined in ChIP-seq peak calling we avoid the term specificity.

## Introduction

Networks of transcription factors, histone modifications and regulatory DNA elements control the spatio-temporal expression patterns of genes during development and in homeostasis. To unravel these regulatory networks and their contribution to developmental processes and human disease, it is imperative to identify the positions of transcription factor binding sites and modified histones throughout the genome. Currently, the most successful approach to identify and map such protein-DNA interactions in vivo on a genome-wide scale is chromatin immunoprecipitation (ChIP) followed by massive parallel sequencing (ChIP-seq) [1]–[3]. In short, ChIP-seq involves cross-linking of DNA and proteins, shearing the cross-linked DNA into fragments and enrichment of DNA bound to the factor-of-interest via immunoprecipitation. Next, these DNA fragments are sequenced, after which reads are aligned to a reference genome and the occurrence of DNA tags is counted. The resulting quantified occurrence of DNA fragments reflects the genomic occupancy by the factor through direct binding or complex formation. Thus, ChIP-seq provides a quantitative map of DNA interaction positions for a given transcription factor, co-factor or modified histone.

In the ideal ChIP-seq experiment there should be no background at all; the presence of reads representing the occurrence of binding at a specific location. However, variability in the affinity of protein-DNA interactions [4] as well as variability due to antibody affinity, sensitivity and specificity, DNA accessibility and chromatin structure [5], differences in exonic and intronic DNA [6], and differences in GC-content [7]–[9], are assumed to generate bias in the observed number of reads and to result in a variable background level within and between ChIP-seq experiments.

These variation sources imply that peak calling requires a computational modelling of tags observed in background regions. A number of peak-calling algorithms have been proposed and implemented. Comparisons of these methods show that different peak-calling methods result in discrepancies in the number and the pattern of identified peaks [10]–[12] and it has to be concluded that no definitive solution for background modelling has been found. Some authors accept that the optimal algorithm may depend on the dataset to be analysed [10] whereas others advise the combination of the outcome of different methods [9], [11]. However, the latter approach can lead to loss of true binding sites [13], which shows that such a combinatorial fusion of several approaches will not always lead to the correct results. Therefore, there is still room for improvement.

Existing peak-calling algorithms, including MACS, CisGenome, PeakSeq, SPP and Sole-Search [14]–[18] compute significance of enrichment relative to local background or combine a global threshold with such a local comparison [19]. This local background is assumed to be variable over the genome but reproducible between replicate experiments. The background is generally determined with so-called Input-seq datasets resulting from sequencing DNA fragments collected without a (specific) immunoprecipitation step. However, it has been shown that Input-seq datasets vary between technical and biological replicates [8] and that these datasets should, therefore, fulfill very strict criteria [20]. Moreover, the use of Input-seq datasets was reported to have only limited advantages [21] and peak calling without Input-seq data has been reported to be at least as effective [22].

In this paper we present some experiments carried out to evaluate the conjectures on which the use of Input-seq datasets are based and to test the usefulness of Input-seq datasets. Based on the results of these experiments we decided to implement a peak-calling system based on background modelling from the ChIP-seq dataset itself. The fraction of the DNA reads in a ChIP-seq dataset that is the result of the immunoprecipitation is reported to be low [23], which means that each ChIP-seq dataset contains a large proportion of background reads. Moreover, ChIP-chip analyses have shown that close to 99% of the arrayed probes do not hybridise [24] which means that ChIP-selected DNA fragments cover only a minor fraction of the total genome length. Following this reasoning, each ChIP-seq dataset contains sufficient background reads to permit modelling of the background present in the dataset. Our peak-calling program, OccuPeak, is based on the abundant presence of these background reads.

We used OccuPeak to test the effects of local versus global background modelling and to test the effect of read density on peak calling. The performance of the algorithm was illustrated by showing its peak-calling consistency in biological replicate datasets. Moreover, the biological relevance of the peaks that were called was evaluated. We chose to use ChIP-seq data generated with the intent to identify regulatory regions active in heart tissue because of the abundance of identified cardiac enhancers (102 in total, Vista enhancer database: http://enhancer.lbl.gov/). To this end, we used data for the cardiac transcription factor TBX3 [25] and the histone acetyltransferase p300 [26] (see Table 1 for an overview of the datasets used).

**Table 1:** Datasets.

ChIP-seq				
Dataset	Study organism	Sequencing platform	Coverage (proportion of genome covered by peaks)	GEO DataSets (GSE)
TBX3 heart over-expression	mouse	AB SOLiD System 3.0	0.0847	GSE44821
p300 heart(1)	mouse	Illumina Genome Analyzer II	0.0494	GSE29184
p300 heart(2)	mouse	Illumina Genome Analyzer II	0.0538	GSE29184
Srf	mouse	Illumina Genome Analyzer	0.0005	GSE21529
Mef2a	mouse	Illumina Genome Analyzer	0.0005	GSE21529
**Input-seq**				
Input control heart(1)	mouse	Illumina Genome Analyzer II	0.0003	GSE29184
Input control heart (2)	mouse	Illumina Genome Analyzer II	0.0003	GSE29184
Input control lung(1)	mouse	Illumina Genome Analyzer II	0.0001	GSE29184
Input control lung(2)	mouse	Illumina Genome Analyzer II	0.0004	GSE29184
**IgG**				
IgG control atria	mouse	Illumina HiSeq 1000	-	GSE46497
IgG control MEL	mouse	Illumina Genome Analyzer	-	GSE49847
**Reference data**				
H3K27ac heart	mouse	Illumina HiSeq 2000	0.0236	GSE49847
DNase1 hypersensitivity sites heart	mouse	Illumina HiSeq 2000	0.0964	GSE40869
Validated enhancers	mouse	-	-	-
Validated cardiac enhancers	mouse	-	-	-
Cardiac gene promoters	mouse	-	-	-

A summary of the datasets used in this study. For ChIP-seq datasets, the sequencing platform, the coverage of the genome by peaks and the GEO DataSets accession number (GSE) is given. The coverage of the genome by peaks is given relative to mappable genome size (1.87 Gb for mm9).

Using ChIP-seq peak-calling software, bench-top biologists, often with little bioinformatics experience, typically encounter a large number of adjustable parameters [10] required to set more or less conservative thresholds [27]. Graphical user interfaces are important to support the analysis needs of such users and to enable them to acquire biological insights [12], [28]. To simplify ChIP-seq data analysis for these researchers, we developed OccuPeak to be a stand-alone ChIP-seq peak-calling program with a user-friendly interface that can serve as a basic research tool.

## Results and Discussion

### Input-seq datasets

Most peak-calling algorithms require input control datasets to model the occurrence of background reads to account for local sequencing and GC-bias. This background is generally modelled with so-called Input-seq datasets resulting from sequencing DNA fragments collected without a (specific) immunoprecipitation step, which is almost as expensive as the ChIP-seq experiment itself. This local background is assumed to be variable over the genome but reproducible between experiments on the same tissue.

#### Correlation between Input-seq datasets

**Figure 1:**
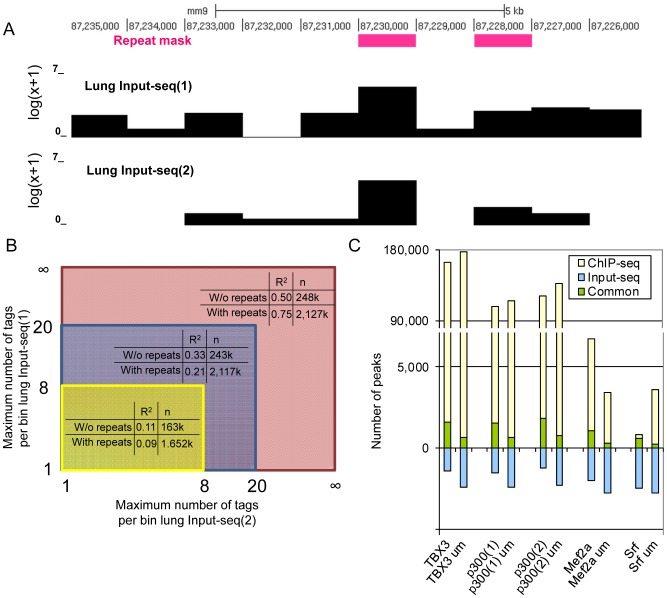
Correlation between Input-seq datasets depends on repeated sequences. **A**. UCSC genome browser snapshot showing tag counts (log scale) in 1 KB bins of two replicate Input-seq datasets. High tag counts are related to annotated genomic repeats. **B**. Correlation between tag counts in two replicate Input-seq datasets for bins without or with genomic repeats (yellow area: bins with tag counts between 1 and 8, blue: between 1 and 20, red: between 1 and infinity). Bins without any tags were excluded from the analysis because they might be the result of unmappable regions. **C**. The small overlap (green) between peaks called in ChIP-seq datasets (yellow) and an Input-seq dataset (blue) is significantly reduced when only uniquely mappable (um) reads are considered in peak calling. This is effect is independent of the number of called peaks.

To determine the extent of the correlation between replicate Input-seq datasets, we divided the genome into bins of 1 kb and counted the number of Input-seq tags in these bins (Figure 1A). At the same time we noted whether these bins overlapped with simple or satellite genomic repeats. Using replicate lung Input-seq datasets, the number of tags indeed correlated (R^2^ bins without repeats  = 0.50, R^2^ bins with repeats  = 0.75; Figure 1B). However, this correlation is largely caused by a limited number of bins with a high number of tags. Excluding bins with more than 20 tags in either dataset, reduced the correlation significantly (R^2^ values of 0.33 and 0.21, respectively); bins with tag counts up to 8 show hardly any correlation (R^2^ values of 0.11 and 0.07, respectively).

The correlation between Input-seq datasets is thus mainly dependent on bins containing a large number of tags. When such tag accumulations are reproducible between Input-seq and ChIP-seq data, these genomic regions are considered to be false positive peaks. To study the extent to which this occurs, we first called peaks on two replicate heart Input-seq datasets and observed approximately 3000 significant peaks in each set. These peaks overlapped for about 78% between the datasets which shows that indeed the most significant regions in these Input-seq datasets are reproducible. To determine the implication of this reproducibility on peak calling in ChIP-seq datasets, we determined the overlap of the peaks called in Input-seq datasets with those called in the TBX3 and the replicate p300 datasets. We found that between 49 and 60% of Input-seq peaks overlapped with ChIP-seq peaks. However, this number only corresponded to between 1–1.5% of the peaks called in the ChIP-seq sets (Figure 1C; green). This implies that the large majority of peaks in these ChIP-seq datasets are located in regions where Input-seq datasets show no correlation. Using tag accumulations in Input-seq datasets to model local background would, therefore, be unjustified.

Mapping artifacts, as a result of genomic repeats, could account for the majority of the overlap observed between replicate Input-seq datasets and between Input-seq and ChIP-seq datasets. Indeed, when we remove those reads that are not uniquely mappable from the ChIP-seq set, approximately 60% of the overlap between Input-seq and ChIP-seq is lost. Such mapping artifacts, however, can easily be detected and avoided before peak calling by using an appropriate alignment setting. This would circumvent the need for Input-seq datasets.

To assess whether the above observations also hold for low-frequency binders we analyzed Srf and Mef2a ChIP-seq datasets [29]. When only uniquely mappable tags were taken into account, OccuPeak called 3408 and 3590 peaks for Srf and Mef2a, respectively, which is a relatively low number and similar to the number of peaks reported by the authors [29]. However, it cannot be determined whether the low number of peaks in these datasets is the result of true low frequency binding or of lower sensitivity ChIP-seq experiments. Regardless, in these datasets we found 6.8% (245) and 8.5% (290) overlap with Input-seq peaks (Figure 1C). Although the overlap with Input-seq data is thus somewhat higher in datasets with a low number of peaks it is still only a minor fraction of the total number of peaks. When all tags were included in the analysis, in both datasets the degree of overlap with Input-seq peaks was significantly higher (Figure 1C). For the Srf dataset the additional noise resulted in a strongly impaired peak-calling power; only 814 peaks were called, of which nearly 70% overlapped with Input-seq peaks. This result is consistent with the observation that mapping artifacts are responsible for most of the overlap between ChIP-seq and Input-seq data.

#### Effect GC-bias on peak calling

The use of Input-seq datasets is also recommended to correct for GC-bias. It has been reported that ChIP-seq reads have a higher average GC-content than the whole genome, possibly due to PCR artefacts [8], [9]. Theoretically, such a bias can result in an overrepresentation of GC-rich regions in peaks being called. However, published data is mainly restricted to the overall distribution of GC-content in ChIP-seq and Input-seq datasets or in whole genomes. Little is known on differences in GC-content between reads occurring in background regions and reads in peak regions. This scarcity of information prompted us to determine the GC-content in different parts of the genome. Furthermore, we determined the GC-content in genome regions covered by single or overlapping reads in ChIP-seq datasets as well as Input-seq sets.

**Figure 2:**
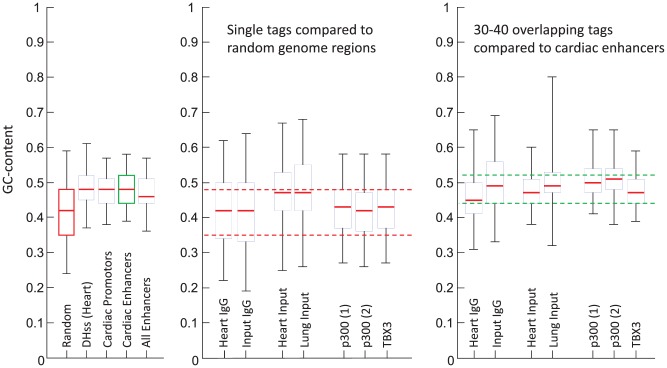
Reviewing evidence of GC-bias in ChIP-seq data. The GC-content was determined for various classes of genomic regions. The GC-content distribution per class is shown in boxplots (whiskers range from 2.5 to 97.5%). **A**. The GC-content distribution of various classes of regulatory elements is plotted next that of random genomic regions (genome background). **B**. The GC-content distribution of genomic regions covered by single tags, resulting from various ChIP-seq experiments, is plotted. The red dotted lines indicate the inter-quartile range of the genome background. **C**. The GC-content distribution of genomic regions covered by tag accumulations (30–40 tags), resulting from various ChIP-seq experiments, is plotted. The green dotted lines indicate the inter-quartile range of validated cardiac enhancers.

DNAse I hypersensitivity sites (DHSs), which are short regions of accessible chromatin characterized by hypersensitivity to cleavage by DNase I, are established markers for regulatory DNA elements [30]. Heart DHSs, cardiac gene promoters, cardiac enhancers and other known enhancers all show on average GC-contents between 47 and 49%, which is higher than the GC-content of random genomic regions of 100 bp in length (Figure 2A). The maximum GC-content of cardiac enhancer and promoter regions, 62%, is far less than the high GC-contents that are reported to result in sequencing bias [31]. Strikingly, single tags in ChIP-seq datasets show a GC-content that is similar to that of random regions (Fig 2B) whereas genomic regions where 30–40 tags overlap, show the high GC-content reminiscent of enhancer regions (Fig 2C). The latter GC-content is similar to the GC-content reported for intron and exon regions [6]. For single tags, the Input-seq datasets show a deviating, higher GC-content whereas an IgG input control (using an irrelevant, non-nuclear antigen) behaves as a ChIP-seq set. The GC-content of tags in the replicate p300 ChIP-seq datasets are indistinguishable from each other. The current finding that IgG input controls show a GC-content for single reads that is similar to those of ChIP-seq datasets would be a point in favour for this kind of input control. Moreover, the significantly higher GC-content in Input-seq datasets shows that the occurrence of reads in these datasets is due to a selection mechanism that differs from the mechanism that is operational in ChIP-seq datasets. The significant difference in GC-contents between ChIP-seq and Input-seq datasets, therefore, makes the latter less appropriate for the modelling of background reads. The similar GC-content of single reads and random genomic regions indicates that ideally background should be estimated from low frequency reads in ChIP-seq experiments.

#### Input-seq datasets can be simulated

**Figure 3:**
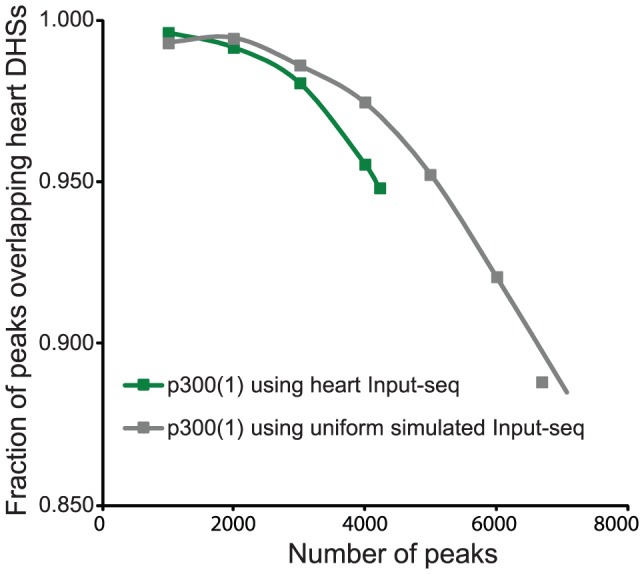
Performance of MACS using Input-seq and simulated input data. MACS was used to call peaks (only chromosome 1) using the p300(1) dataset. Heart Input-seq data or a simulated uniform background dataset were used as input control. The influence of the input control set on peak-calling performance was measured using overlap with DHSs as outlined in the legend of Figure 8.

The observation that single reads show the GC-content of random regions suggests that single reads in ChIP-seq datasets occur randomly in the genome. This implies that background can be modelled on these low frequency reads. To test this hypothesis, we simulated a background set with a random-uniform background and used this set as an input control set. We used this simulated and an actual Input-seq dataset to compare the results on peak calling on the p300(1) dataset with the peak-calling program MACS (Figure 3).

The number of DHSs reported from heart tissue exceeds 260,000 (∼10% genome coverage) and exceeds the number of peaks called from ChIP-seq datasets. Therefore, the DHS dataset can be used as a reference set to determine the positive predictive value of a peak-calling algorithm, i.e. its ability to call peaks representing true binding sites. For the 4000 most significant peaks in each set, more overlap with cardiac DHSs was found for the peak-set based on the simulated input control data (Figure 3; 97.5% and 95.5%, respectively; p<0.001). This showed that in terms of biological relevance, peak calling by correcting for a uniform background is at least as effective as using a local window and an Input-seq dataset to correct for background bias.

#### Fraction of tags associated with peaks

Replacing the use of Input-seq datasets with background modelling based on the ChIP-seq dataset requires that background information is present in the ChIP-seq dataset itself. It has been reported that the fraction of reads associated with specific immunoprecipitation in ChIP-seq is low [23]. Indeed for the TBX3 data, only 57% of the tags (13,088,900 in total) were associated with called peaks; for the p300 data the percentages were 38% (replicate 1) and 29% (replicate 2). The genomic coverage of peaks ranges from 5% to 8% for TBX3 and the p300 replicate datasets, implying that over 90% of the genome is available for background modelling. These results indicate that each ChIP-seq dataset contains sufficient background reads to model the background present in the dataset.

### OccuPeak model

The above experiments showed that correlation between replicate Input-seq datasets results mainly from high frequency, repeated, regions whereas only a small fraction of these regions overlapped with significant regions in a ChIP-seq experiment. Moreover, equally effective peak calling could be accomplished after modelling a uniformly distributed background. The observation that single reads show the same GC-content as randomly sampled regions suggests that the low frequency reads in ChIP-seq datasets represent the background noise. Because a large proportion of the genome in ChIP-seq experiments is covered by such abundant background reads we propose to model the background required for peak calling from the ChIP-seq experiment itself.

Therefore, we propose a background model that assumes that every location on a chromosome has the same chance to be covered by a background tag in the ChIP-seq dataset. The number of regions covered by at least a single tag in the dataset can then be modelled as N(1) = p.A, where A is the length of the mappable genome and p is the probability of a tag being observed. When all tags are independent, the chance of two tags overlapping at the same position in the genome is the product of the probability of a single occurrence. So, when all tags are observed by chance only, the number of regions with at least n overlapping tags, N(n), is given by our background model:



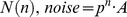
( Eq. 1)


The observed number of regions with n or more overlapping tags is a combination of background and specific occurrence of tags. Therefore, to fit this noise model to the actual observed tag distribution, an offset representing the number of real peaks (B) has to be included. The cumulative distribution of the number of regions with n or more overlapping tags in a ChIP-seq dataset can thus be modelled as:



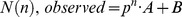
( Eq. 2)


This model can be fitted to the observed counts, N(n), of regions covered by at least n tags, for n is 1 to 4, to estimate the model parameters p, A and B. With these parameters, the expected occurrence of regions of at least n tags due to background tags can be calculated with Eq. 1. The ratio of the observed N(n) and the expected N(n) due to noise is then defined as the excess ratio (ER) for each n:



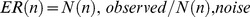
( Eq. 3)


When significantly more regions with a given number of tags are observed than expected, these regions are no longer considered to be background and thus should be called real peaks. For convenience, log10(ER) is used to set this significance threshold. By default a threshold value of 2, equivalent to an excess ratio of 100, was applied. The threshold level of ER has to be set by the user to account for technical and biological variability.

### OccuPeak performance

#### Local background modelling

**Figure 4:**
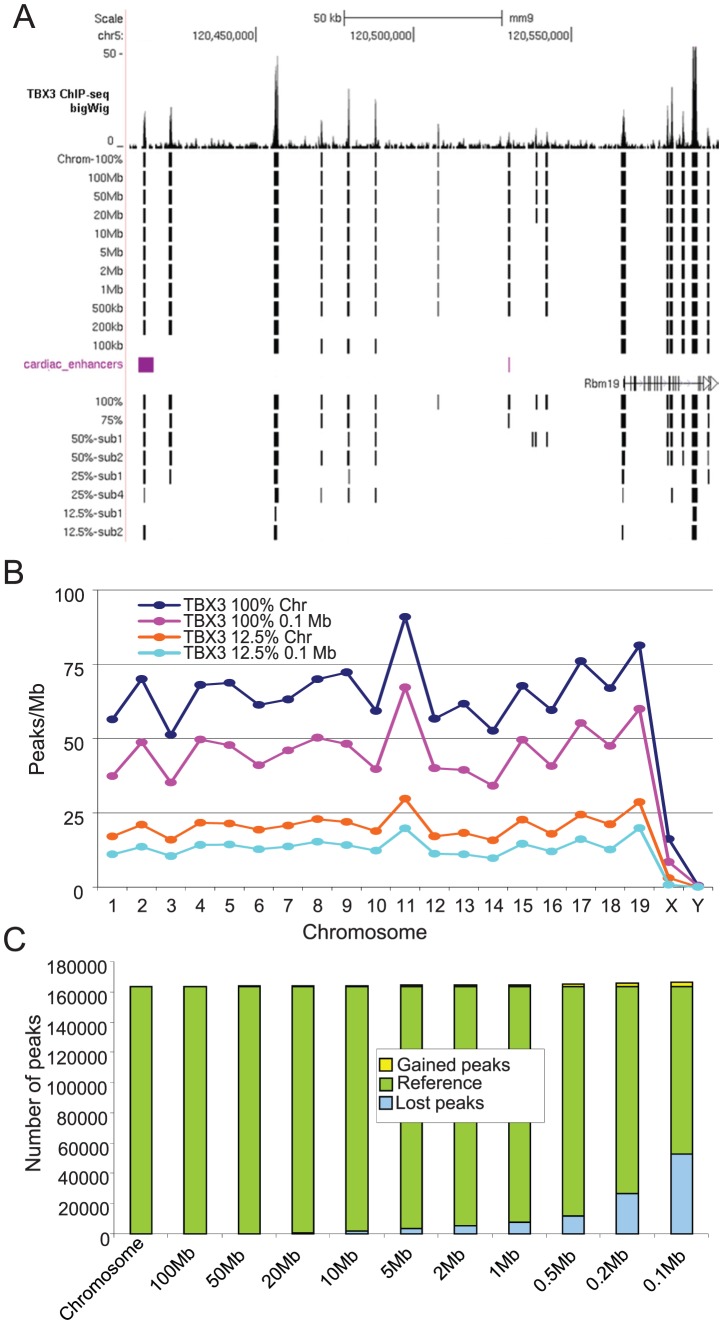
Effect of window size and tag density on the pattern and number of called peaks. Peaks were called with OccuPeak in the TBX3 ChIP-seq dataset using different window sizes and tag densities. **A**. UCSC genome browser snapshot capturing the effects on peak calling in a region containing 2 validated cardiac enhancers. **B**. Mean number of peaks called per Mb of genome. Note the (almost perfect) parallelism of the profiles for different tag density (100% and 12.5%) and window size (chromosome and 0.1 Mb). **C**. Effect of window size on the gain or loss of peaks. When the peaks called with a chromosome-wide window are used as a reference (green), smaller windows lead to loss of peaks (blue) but hardly ever to gain of peaks (yellow).

Most peak-calling programs use sliding windows to determine the abundance of local background tags to be used as a local peak-calling threshold [12]. Moreover, the performance of ChIP-seq peak-calling methods has been reported to depend on the total number of reads, i.e. read density, in the dataset [27]. To investigate whether those issues affect the performance of the OccuPeak algorithm, the effect of the size of the sampling window and of the tag density on the number of peaks and the pattern of peaks was determined. To this end, systematic sub-sampling was used to generate ChIP-seq datasets containing 12.5, 25, 50 and 75% of the total number of tags. For each subset, OccuPeak was applied with window sizes ranging from 0.1 Mb to complete chromosomes. The required number of windows to completely cover each chromosome was distributed uniformly with minimal overlap. The resulting peak sets were visualized and compared (Figure 4A; File S1).

When significant differences in background exist, the application of the OccuPeak model using small 0.1 Mb windows should result in improved peak calling. This difference would be reflected in the number of peaks called per window and the occurrence of a different pattern of peaks. However, this analysis showed that only the number of called peaks decreases whereas the pattern of observed peaks per chromosome does not change with the use of small sampling windows (Figure 4B). The profile of the average number of peaks per Mb genome of each chromosome is not dependent on window size (Figure 4B) although the total number of peaks decreased between chromosome-sized windows and 0.1 Mb windows (Figure 4B, top). The pattern of peaks correlates significantly between chromosome-wide and 0.1 Mb windows (R^2^ = 0.96). The loss of peaks reflects the decreasing power to call peaks when fewer tags are available. Indeed, in a 12.5% sample of the same dataset, the number of peaks called is also substantially lower but the pattern of peaks over the genome is unaffected (Figure 4B, bottom).

To identify which peaks are either gained or lost using smaller windows we compared overlap between the peaks resulting from the use of the chromosome-sized windows with the peaks observed with decreasing window sizes. Peaks are gained relatively rarely, with a maximum of less than 2% of the peaks using the smallest 0.1 Mb windows (Figure 4C; yellow). However, loss of peaks becomes more frequent as the window size decreases (Figure 4C; blue). These 'missed' peaks are generally associated with relatively low tag counts and thus represent less significant binding regions. Similarly, peaks are missed when sub-samples of the ChIP-seq dataset are analyzed (Figure 4A and 4B) illustrating the decreased peak-calling power of small datasets [27].

To determine biological significance of these subpopulations of peaks we determined the positive predictive values by looking at overlap with cardiac DHSs. Peaks that were observed with both the chromosome-sized and the 0.1 Mb windows, overlap for 78% with cardiac DHSs whereas the peaks missed in the 0.1 Mb set show a significantly lower 60% overlap with DHSs (p = 0.001; Z-test). However, the peaks that are gained with the 0.1 Mb windows only reach 39% overlap (p<0.001, compared to both other categories). This result also shows that peak-calling performance is not improved by local background modelling. The use of a small window size to account for local variation in background is therefore not recommended.

#### Consistency of peak calling between replicate datasets

**Figure 5:**
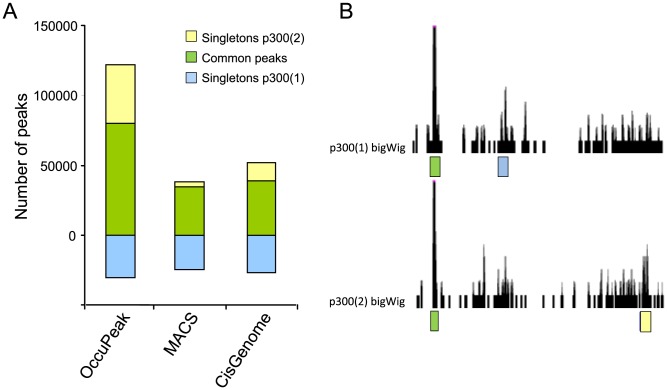
Consistency of different peak-calling methods. OccuPeak, MACS and CisGenome were used to call peaks for each of the two replicate p300 ChIP-seq experiments generated by the ENCODE consortium (GSE29184). **A**. Peaks are considered common (green) if they were identified in both replicates and singleton if they were only found in the current replicate (yellow and blue), as depicted in the UCSC genome browser example (**B**).

The availability of replicate p300 ChIP-seq experiments [26] provided the opportunity to determine the consistency of peak-calling algorithms between biological replicates. Peaks were considered common (Fig 5; green bars) if they were identified in both replicate datasets and singleton if they were only identified in one replicate set (Fig 5; blue and yellow bars for replicate 1 and 2, respectively). Occupeak found 52% peaks common to both datasets (Figure 5; bar 1). We also determined the consistency in peak calling for the MACS and CisGenome algorithms (Figure 5, bars 2 and 3). Cisgenome showed 50% of peaks being called consistently between sets, whereas MACS reached 54%. However, peak-calling power, reflected in the number of peaks called at default threshold, differs per method: the number of common peaks identified by OccuPeak exceeds the total number of peaks called by the other peak callers. Although the different peak-calling methods do not differ in consistency of peak calling, an analysis based on overlap between datasets will benefit from a large number of observed peaks because it avoids the loss of information when datasets differ substantially in read density or background noise.

### Calling biologically relevant peaks

#### Peak-calling power and sensitivity: cardiac enhancers

**Figure 6:**
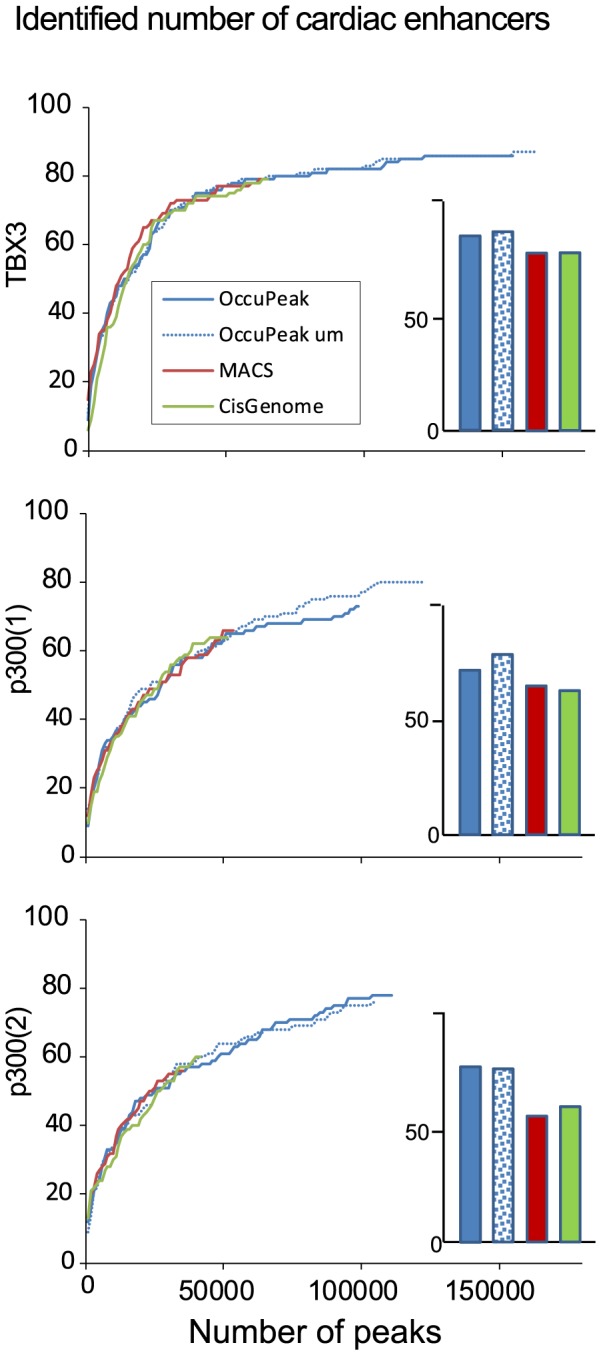
Biological Validation: overlap with cardiac enhancers. OccuPeak, MACS and CisGenome were used to call peaks from the TBX3 and the two replicate p300 ChIP-seq datasets. Peaks were then sorted on peak significance and overlap with cardiac enhancers was determined. For visualization, the number of most significant peaks was incremented in steps of 1000 peaks. A set of 102 validated cardiac enhancers was used to assess the sensitivity of the peak-calling method and the biological relevance of the called peaks. The number of enhancers identified using the default threshold of each peak calling method is plotted in the bar graphs.

**Figure 7:**
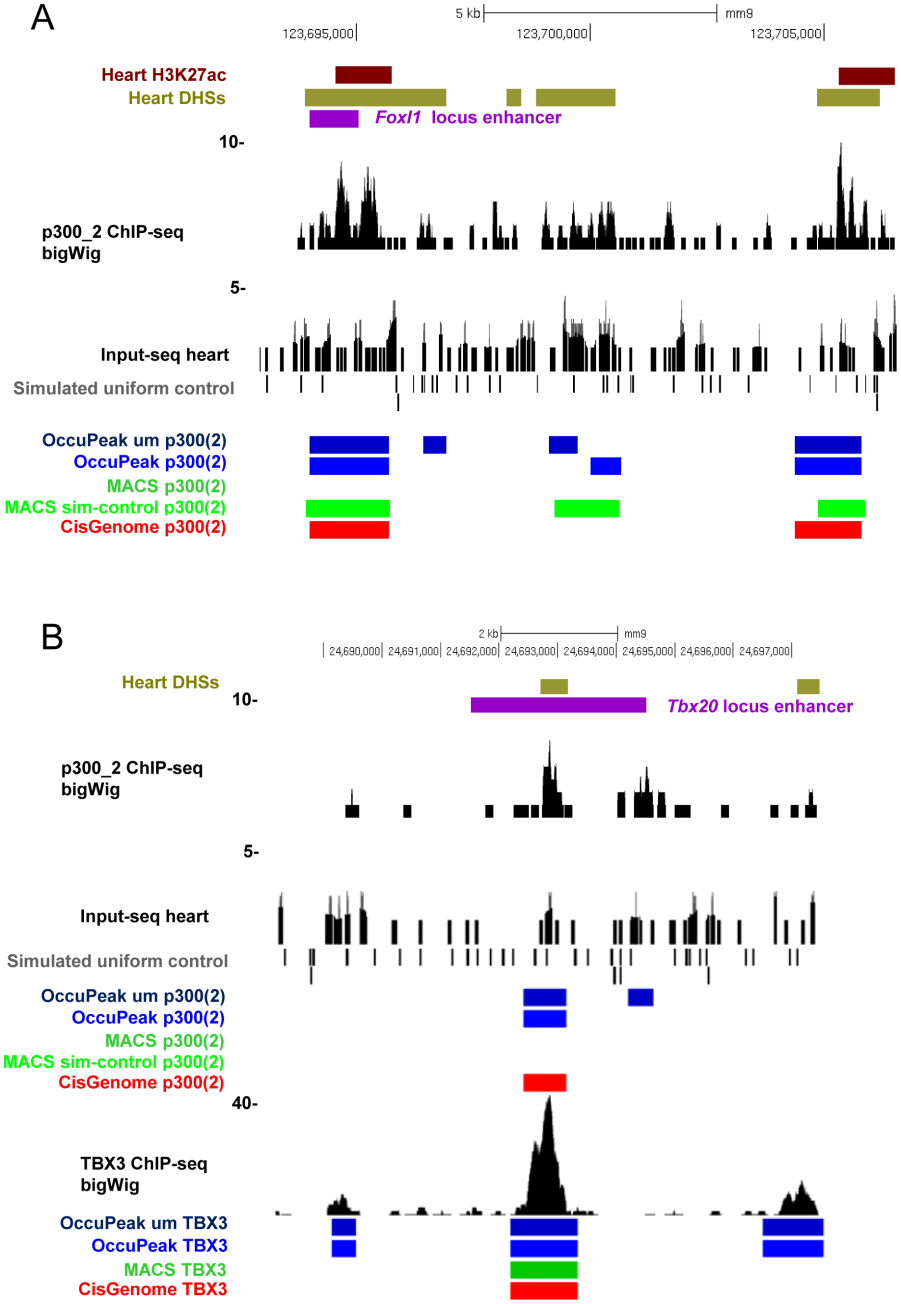
Visualization of overlap analysis. Visual inspection with the UCSC genome browser can show where and why certain enhancers are missed by a particular peak-calling method. **A**. Relatively small local increases in input control tag density can result in a locally decreased sensitivity of the method. An enhancer on the Foxl1 locus is missed by MACS when heart Input-seq data is used as input control, but detected when a simulated uniform dataset is used as control instead. **B**. Similarly, an enhancer located on the Tbx20 locus is missed by MACS when an input control is used on the p300(2) data. When applying the same input control on the more abundant TBX3 data, the enhancer is marked by all methods. Abbreviations: um  =  dataset in which only unique tags are mapped; sim-control  =  dataset where simulated uniform data is used as input control for peak-calling.

Overlap with validated cardiac enhancers can be used to assess the biological relevance of an identified set of peaks. To this end we used a set of validated cardiac enhancers that consists of 102 mouse genomic regions that have reproducibly been shown to drive cardiac reporter gene expression in transgenic mouse embryos. Overlap analysis was carried out with peak sets called by OccuPeak, CisGenome and MACS (Figure 6). Figure 7 shows an example of a UCSC session with detailed results. When analyzing the TBX3 ChIP-seq set, OccuPeak identified 86 enhancers (84%; Figure 6; bar graphs). MACS and CisGenome both called fewer peaks, identifying 79 enhancers. For the replicate p300 ChIP-seq datasets, OccuPeak identified 73 enhancers in replicate 1 and 78 in replicate 2. MACS and CisGenome identified 66 and 64 enhancers, respectively, in replicate 1 and 56 and 60 enhancers, respectively, in replicate 2. In all cases OccuPeaks performance increased when only uniquely mappable tags were considered. Taken together, the default thresholds used by CisGenome and MACS lead to impaired peak-calling sensitivity compared to OccuPeak. Especially for the p300(2) dataset this conservative threshold leads to a significant reduction in identified cardiac enhancers (Figure 7).

One can argue that the total number of enhancers that is correctly identified is biased by the total number of peaks that is called. To address this argument, the threshold setting of each individual peak-calling method was stepwise adjusted until the same number of peaks was called at each step. This iterative approach shows the relationship between peak-calling power (number of peaks; X-axis) and sensitivity (number of identified cardiac enhancers; Y-axis) that is unbiased by the difference in total number of peaks (Figure 6). The general shape of the resulting curves is biphasic, showing a sharp increase of identified enhancers for the most significant peaks followed by a steady increase towards a plateau in sensitivity. This relation holds for the TBX3 data as well as both p300 datasets. Statistical comparison of the number of identified enhancers at the maximum shared number of peaks showed that there was no significant difference between any of the methods (p>0.77). This leads to the conclusion that the ability of OccuPeak, not requiring an Input-seq dataset, to identify validated cardiac enhancer sites is similar to that of other methods when a limited number of peaks is called.

#### Positive predictive value of peak calling

**Figure 8:**
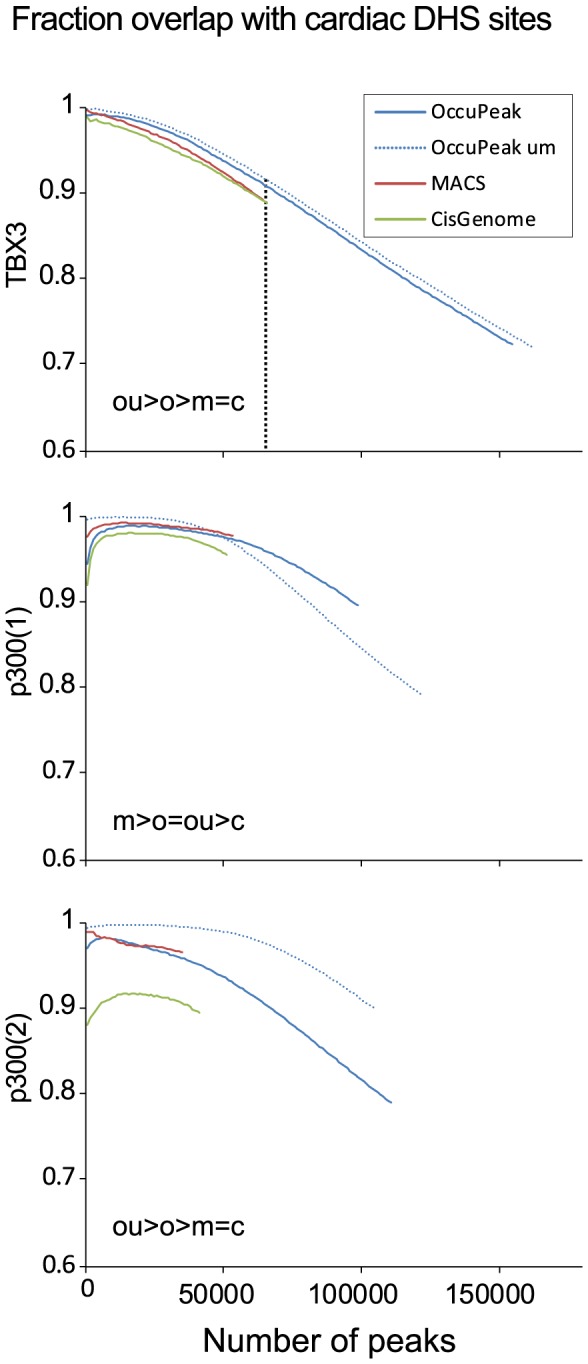
Biological Validation: overlap with cardiac DHSs. OccuPeak, MACS and CisGenome were used to call peaks from the TBX3 and the two replicate p300 ChIP-seq datasets. Peaks were then sorted on peak significance and overlap with cardiac enhancers was determined. For visualization, the number of most significant peaks was incremented in steps of 1000 peaks. Overlap of peaks with DNaseI hypersensitivity sites (DHSs) found in heart tissue was used to assess the positive predictive value of the peak-calling methods. In the p300(2) dataset the performance of OccuPeak was significantly better when only uniquely mappable tags were considered. The results of the statistical comparison at the maximum common number of peaks (vertical dotted line) is given as a string in which ' = ' indicates that the overlap is not significantly different between the methods and '>' that the overlap differs significantly at p<0.0001 or less (O = OccuPeak, all reads; OU = OccuPeak, uniquely mappable reads; M = MACS; C = Cisgenome).

We determined the ability of the different peak-calling methods to call peaks representing true binding sites, i.e. their positive predictive value, using overlap with DHSs as marker for regulatory DNA. Overlap analysis of ChIP-seq peaks with DHSs showed that a high percentage of peaks is associated with a DHSs (Figure 8). Strikingly, irrespective of the dataset and peak caller used, the top 10,000 most significant ChIP-seq peaks showed close to 100% overlap with DHSs. The degree of overlap with DHSs dropped with the increasing number of less significant peaks. However, the overlap of peaks with DHSs did not drop below 72% for TBX3 and 79% for the p300 replicate sets, even with the large number of peaks called with the default setting of OccuPeak. Statistical comparison at the highest common number of peaks of the performance curves showed that for peaks called by OccuPeak in the TBX3 ChIP-seq data, the overlap with DHSs is significantly higher than for peaks called by each of the other peak callers (Figure 8). For the p300 datasets this test showed that, depending on the dataset either MACS or OccuPeak performed best whereas CisGenome performed significantly worse in both sets. Overall OccuPeak performs better or equally well compared to other peak callers in calling peaks that overlap with regulatory DNA and is thus likely to call peaks that represent binding sites without the need for input control datasets.

#### Peak-calling power and sensitivity: H3K27ac

**Figure 9:**
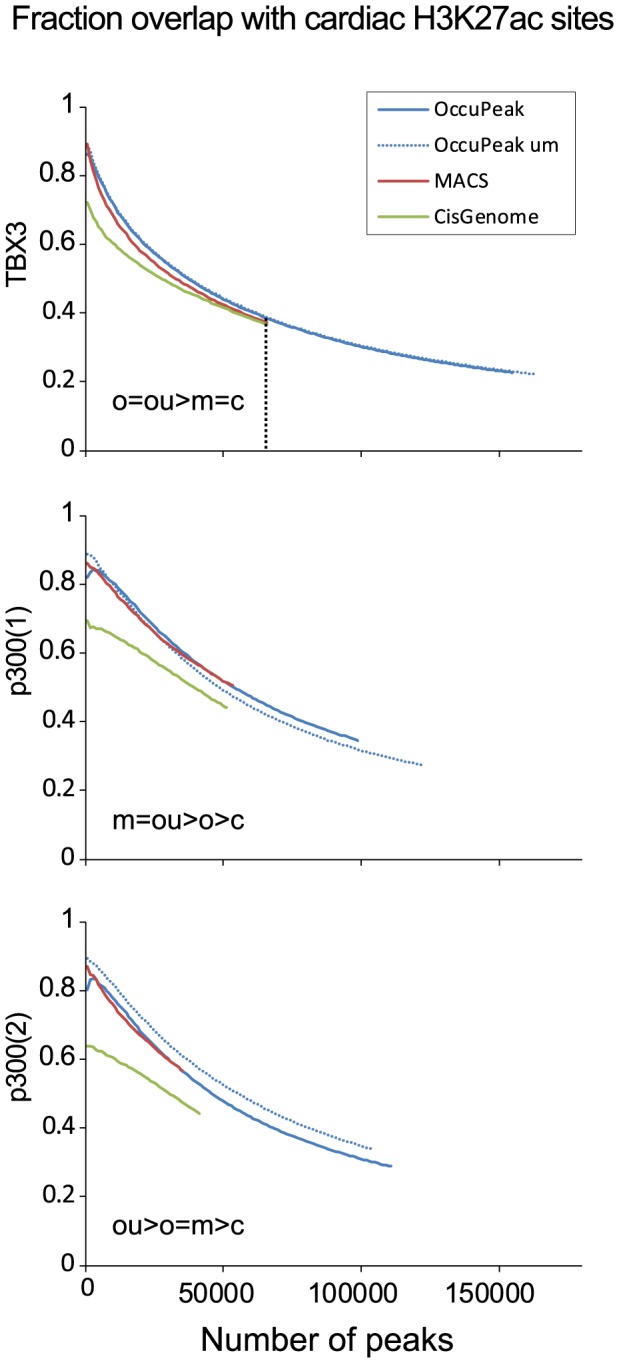
Biological Validation: overlap with cardiac H3K27ac sites. OccuPeak, MACS and CisGenome were used to call peaks from the TBX3 and the two replicate p300 ChIP-seq datasets. Peaks were then sorted on peak significance and overlap with cardiac enhancers was determined. For visualization, the number of most significant peaks was incremented in steps of 1000 peaks. Overlap of peaks with H3K27ac sites was assessed as measure for active enhancers. In the p300(2) dataset the performance of OccuPeak was significantly better when only uniquely mappable tags were considered. The results of the statistical comparison at the maximum common number of peaks (vertical dotted line) is given as a string in which ' = ' indicates that the overlap is not significantly different between the methods and '>' that the overlap differs significantly at p<0.0001 or less (O = OccuPeak, all reads; OU = OccuPeak, uniquely mappable reads; M = MACS; C = Cisgenome).

H3K27ac is a marker reported to distinguish active enhancers from poised or inactive enhancers [32]. Here, we used a cardiac specific H3K27ac dataset in which 44044 regions were marked covering approximately 2.4% of the genome [26]. The most significant peaks called by OccuPeak and MACS reach an overlap of approximately 90% with H3K27ac sites whereas CisGenome reaches approximately 70%. Statistical comparison at the last common point of the performance curves (Figure 9) showed that peaks called by OccuPeak (for all and for only uniquely mappable reads) in the TBX3 dataset had significantly more overlap with H3K27ac sites than those called by other methods. However, in the p300 replicate sets MACS and OccuPeak performed similarly but the restriction to uniquely mappable had a different effect in each of these sets. Both methods showed a significantly higher overlap with H3K27ac sites than CisGenome for the p300 datasets. This overlap analysis thus showed that the ability of the default setting of OccuPeak to identify active enhancers is similar to MACS and better than CisGenome.

#### Association with cardiac GWAS SNPs

The genome of each individual contains many single-nucleotide variants (SNPs) that are associated with disease susceptibility. Recent estimations indicate that ∼90% of disease and trait-associated variants occur within non-coding sequences, a large number of which may correspond to regulatory elements [30], [33], [34]. To further validate the biological relevance of peak-calling, we assessed whether the cardiac TBX3 and p300 ChIP-seq peaks called by OccuPeak were enriched by SNPs associated with cardiac function. To this end, we assembled 42 such SNPs from major genome wide association studies (GWAS) [35]–[39]. A control SNP set was created by randomly selecting 504 SNPs, not associated with biological function [40], within 1 Mb of known UCSC genes. This 1 Mb genomic distance cut-off was taken based on multiple studies using 3C-derived technologies which reveal that meaningful chromatin interaction is confined to topological domains of roughly 1 Mb [41]–[43]. As TBX3 is an important cardiac transcription factor [44], [45], we asked whether we could establish a relationship between the presence of TBX3 binding-sites and SNPs associated with cardiac function. Lacking human TBX3 ChIP-seq data and taking into account the evolutionary conservation of the TBX3 protein, we used a comparative genomics approach. To enable overlap analysis, we lifted-over the called ChIP-seq peaks from the mouse genome (mm9) to the human genome (hg18) using the Galaxy interface, applying a 0.6 minimum ratio of bases that must remap, without allowing for multiple output regions. The peak-sets that OccuPeak identified for TBX3 and both p300 replicates were all significantly enriched with SNPs associated with cardiac function (Table 2 & 3). This result supports the conclusion that OccuPeak uncovers binding sites that are enriched with functionally relevant regulatory regions. Furthermore, similar to the findings of numerous studies [46]–[49], this result indicates that a substantial part of these regulatory regions are conserved across evolution from mouse to human and can therefore be of potential clinical relevance.

**Table 2:** Association with cardiac GWAS SNPs.

	mm9 peaks (OccuPeak)	Hg18 peaks (OccuPeak)	Cardiac SNPs (42)	Control SNPs (504)	Significance
**TBX3**	163699	99414	8	16	2.19E-05
**p300 replicate 1**	108397	70949	4	12	8.37E-03
**P300 replicate 2**	121491	75232	8	15	2.65E-05

Peaks called by OccuPeak for TBX3 and both p300 replicate datasets were converted from the mouse genome (mm9) to the human genome (hg18) to enable overlap analysis with known human SNPs. A two-sample Z-test was performed to test whether called peaks overlap more frequently with SNPs associated with cardiac function than with control SNPs [40]. Control SNPs were randomly selected from a population of SNPs not significantly associated with any GWAS signal and located within 1 Mb of known UCSC genes. SNPs associated with TBX3 peaks are listed, including their phenotype. Further details and references are in the Results section of the main text.

**Table 3:** List of cardiac GWAS SNPs associated with TBX3 peaks.

snp	effect	Locus	ref
rs3807989	Prolongued PR-interval & increased AF risk	CAV1	[35]
rs12053903	Shortened QT-interval	SCN5A	[39]
rs3922844	Prolongued PR-interval	SCN5A	[35]
rs6801957	QRS duration	SCN10A	[36]
rs11677371		MEIS1	[40]
rs4433986		MEIS1	[40]
rs7312625	Shortened PR-interval	TBX5	[39]
rs1895585		TBX5	[40]

The overlapping SNPs and their reported effects and locus are given.

## Concluding Remarks

The use of Input-seq datasets by most peak-calling programs assumes these datasets to represent a reproducible occurrence of background reads. However, we found that the most significant correlation between Input-seq datasets occurs in the regions with highest tag counts which are often associated with genomic repeats. Even then, only about 1% of the peaks called on ChIP-seq datasets overlap with peaks in Input-seq datasets; this overlap could be halved when reads associated with repeats were excluded. Bias in peak calling due to reproducible background can thus easily be reduced by considering uniquely mappable reads only.

The current analysis shows that the GC-content of regulatory genomic regions is much lower than the GC-content at which significant sequencing bias occurs [31]. We show that single tags in Input-seq datasets have a higher GC-content than single tags in ChIP-seq datasets but that the latter share their GC-content with randomly generated reads. This, and the observation that a dataset with simulated uniform background noise can be used for effective peak calling, supports the basic assumption of OccuPeak that the abundant single tags represent background reads and can thus be used to model the background in ChIP-seq datasets.

With OccuPeak we showed that background modelling based on chromosome-wide windows gives a better peak-calling result with a higher positive predictive value than background modelling based on local windows. Local background modelling, which is used by most other peak-calling programs, makes that the peak-significance is dependent on the local tag distribution. In contrast, the measure of peak significance used by OccuPeak, the excess ratio, is only dependent on the read density of a peak and the global noise level. The interpretation of the significance of a peak is, therefore, independent of its location in the genome.

OccuPeak's ability to identify known cardiac enhancers was similar to other methods. The analysis of overlap with cardiac DHS and H3K27ac sites demonstrated that OccuPeak calls a larger number of peaks with similar or even significantly more overlap compared to MACS and CisGenome. The performance of OccuPeak could be further increased when only uniquely mappable reads are considered. Furthermore, peaks called by OccuPeak were significantly enriched in SNPs that are associated with cardiac function. These analyses lead us to conclude that the use of OccuPeak results in the identification of biologically relevant peaks from ChIP-seq datasets.

CisGenome and the Galaxy implementation of MACS are relatively user-friendly but the majority of peak-calling methods is exclusively command line based which reduces their accessibility for the basic researcher. We developed OccuPeak to be a user-friendly alternative to existing ChIP-seq peak-calling applications. The use of standard file formats allows its inclusion into existing data analysis pipelines. OccuPeak does not require user settings, except for the peak-calling threshold, which simplifies, and standardizes the analyses. The stand-alone program is made available for the scientific community (http://occupeak.hfrc.nl).

The novelty of OccuPeak lies in the fact that it directly couples background modelling and peak calling. The current experiment was set up to determine whether such modelling of background tags should be local or global, to determine its consistency and effectiveness in peak calling and to compare this performance to peak calling based on Input-seq data. The results show that peak calling without an Input-seq control dataset is at least as powerful and sensitive, and often more biologically relevant, than other peak callers. OccuPeak thus successfully circumvents the need of Input-seq datasets, which reduces experimental costs, without compromising experimental accuracy.

## Material and Methods

### Datasets

To evaluate the performance of OccuPeak and to compare it to the performance of other peak-calling methods, we used ChIP-seq datasets originally generated with the purpose to identify putative cardiac enhancers across the genome (Table 1). These sets are 1) TBX3 ChIP-seq data from the adult male mouse heart over-expressing TBX3 [25], which was generated for this study on the ABI SOLiD sequencing platform (GSE44821) and 2) two replicate p300 ChIP-seq experiments with adult mouse hearts generated by the ENCODE consortium [26] (GSE29184) and 3) Srf and Mef2a ChIP-seq data generated by the laboratory of William Pu [29] (GSE21529). We processed all ChIP-seq datasets starting with the raw reads.

For the comparison with MACS and CisGenome we used the heart Input-seq dataset provided by the ENCODE consortium [26] (GSE29184). To study whether and how the use of Input-seq data affects the performance of MACS, we generated simulated background sets as alternative input control sets. In the simulation of a background set every location on the chromosome had the same chance to occur randomly as a tag. For accurate comparison, the number of simulated tags was set to be equal to the number of tags present in the cardiac Input-seq dataset. The simulation was performed using the ‘runif’ function of R (version 2.15.2), which randomly generates genomic coordinates at which simulated tags were placed.

### Methods: Overlap analysis

Overlap between peaks or between peaks and SNPs, DHSs, H3K27ac sites or known enhancers was defined as at least a single overlapping genome coordinate. Where the performance of the peak callers was compared by overlap with DHS sites, H3K27ac sites or known enhancers we corrected for differences in peak width. To this end we created a set of merged peaks which extended the total genomic coordinates of the peaks called by the different peak callers. Of each merged peak was noted by which peak callers it was called and with which significance. When more than one peak overlapped with a single merged peak, the most significant value was assigned.

### Methods: Peak calling

#### Raw sequence reads: SRA and FASTQ processing

The sequence reads generated by sequencing platforms are in various forms of the FASTQ format. FASTQ is a text-based format for storing both a base pair sequence and its corresponding quality scores [50]. By convention, the raw data from ChIP-seq experiments on Geo DataSets are available in Sequence Read Archive (SRA) format. FASTQ and SRA are analogous formats and the open source SRA Toolkit software package (http://www.ncbi.nlm.nih.gov/books/NBK56560/) can be used to convert between these formats. The Galaxy software interface (https://main.g2.bx.psu.edu/) only accepts raw data from sequencing platforms in the FASTQ format. For further use in any downstream Galaxy applications, the FASTQ file needs to be groomed to the default Sanger FASTQ format. For this we used the FASTQ Groomer (version 1.04) available on Galaxy [51].

#### Mapping the reads: Bowtie

Bowtie (version 1.1.2) [52] was used to map reads to the reference genome, in this case the mouse genome (mm9). We used a seed length of 28 and a maximum number of 2 mismatches allowed within the seed. The '- - best' option was used to ensure that only the best alignments, in terms of number of mismatches and read quality, were reported by Bowtie when multiple reads were mapped to the same genomic location. The '-k' option was set to 1 to ensure that only 1 valid alignment was mapped per singleton read in case that a read was reported to have several valid alignments to the reference genome. In such cases the first valid alignment Bowtie encounters was chosen. Alternatively, the '-m' option was set to 1 to review peak calling without the influence of repeats. Using this setting, all alignments for a read are suppressed if more than 1 reportable alignment exists across the genome. For the remaining parameters the default settings were used. Mapping with Bowtie, results in a Sequence Alignment Map (SAM) file. PCR duplicates, which may introduce bias, can be removed using the ‘remove duplicates’ function of OccuPeak. In the described application of the OccuPeak pipeline PCR duplicates were not removed; we consider the use of the Rmdup tool optional.

#### Methods: Peak calling with MACS

The Model-based Analysis of ChIP-seq (MACS) package [14] uses tag shifting and sliding windows to scan chromosome regions for the presence of peaks. A dynamic Poisson distribution is applied to model the local background signal. We used MACS version 1.4.0rc2 as available on the Cistrome server (http://cistrome.org/ap/). BAM files were used as input. We ran MACS with Input-seq data. For peak calling, effective genome size was set to the value applicable for the mm9 genome (corresponding to 1.9 Gb) and tag size was set as defined in the BAM files; default values were set for the remaining parameters.

#### Methods: Peak calling with CisGenome

CisGenome [15] requires an Input-seq dataset to perform the recommended two-sample peak calling. CisGenome uses sliding windows to scan the genome to count the number of ChIP-seq and Input-seq tags and a binomial distribution is estimated from the Input-seq data. We used the Galaxy Text Manipulation toolset to convert SAM files into the ALN format (http://www.biostat.jhsph.edu/~hji/cisgenome/index_files/tutorial_seqpeak.htm) required by CisGenome. For peak calling the default parameters of the CisGenome program were used.

### Methods: Statistics

Differences in overlap of peaks with known enhancers, DHSs and H3K27ac sites, as well as their enrichment with cardiac GWAS SNPs, was determined using the two-sample Z-test implemented in the SAGEstat program [53].

### OccuPeak implementation

OccuPeak accepts Sequence Alignment Map (SAM) files as input. These SAM files were generated using the pipeline presented in the Methods and are the default output of the Bowtie mapping program. The output of OccuPeak is a file in BED format which is compatible with the UCSC genome browser (http://genome.ucsc.edu/).

#### DNA fragment reconstruction

Sequencing of sheared DNA fragments results in reads that are typically much shorter than the original fragments. When reads are aligned to the genome, tags from the forward strand typically appear shifted in 5'-direction compared to those from the reverse strand. Therefore, the tags from both strands have to be extended in their 3′-direction to the estimated original fragment length [20], [54]. To this end, we determined continuous regions for each strand separately and those regions that uniquely overlap between the forward and reverse strand were selected. Regions with log(ER)>50 were excluded because they might result from sequencing or alignment artefacts. The distance between the midpoints of the 200 most significant overlapping forward and reverse regions was determined. The median of these distances was used as an estimate of the average length of the DNA fragments. This length was applied to extend the tags from each of the strands separately in the 3'-direction. Then the tags from both strands were merged and peaks were identified in the merged dataset.

#### Output of OccuPeak

OccuPeak writes the identified peaks to a text file in BED format (http://genome.ucsc.edu/FAQ/FAQformat.html#format1). The header of this file contains information on the reconstructed fragment length and applied ER threshold. In the body of the file, the first three columns give chromosome, start coordinate and end coordinate of a region that is identified as a peak. The fourth BED column contains the surface area of the peak in bp, calculated as the sum of the lengths of the overlapping tags in this region. The fifth column reports the corresponding log(ER) value of the peak. Columns 6, 7 and 8 are not used. Column 9 contains RGB values corresponding to user defined settings to distinguish different ER categories which can be used in the UCSC browser to distinguish categories of peaks. Optionally the OccuPeak program can save a summary file in which the model parameters, genome coverage and number of peaks are reported for each sampling window or chromosome.

### OccuPeak availability

The peak-calling algorithm that is based on the OccuPeak background model was implemented in Matlab version 2012b (The MathWorks, Inc., Natick, Massachusetts, USA) and was compiled into stand-alone programs for the Windows and Linux environments. In both environments Occupeak runs as a stand-alone application OccuPeak can be downloaded from (http://occupeak.hfrc.nl). To run the program the freely available Matlab Component Runtime environment, which comes with an automatic installation, also needs to be installed (http://www.mathworks.nl/supportfiles/MCR_Runtime/). The OccuPeak package is self-extracting and will automatically generate the directory structure that the program needs. For the mouse genome (version 9; mm9), a text file listing chromosome lengths is included in the OccuPeak package. For other genomes, a file containing the lengths of chromosomes can be downloaded from the UCSC genome browser database and placed in the designated directory.

Source code is available under a BSD license (http://occupeak.hfrc.nl).

## Supporting Information

File S1 A supplemental UCSC genome browser session has been made accessible, enabling genome browser inspection of the results generated in this study.
(PDF)
