# Supplementary material for: OccuPeak: ChIP-Seq Peak Calling Based on Internal Background Modelling
Source: PLoS One. 2014 Jun 17;9(6):e99844. doi: 10.1371/journal.pone.0099844 (PMC4061025; doi:10.1371/journal.pone.0099844)
Supplement: File S1 — A supplemental UCSC genome browser session has been made accessible, enabling genome browser inspection of the results generated in this study. (PDF) [file pone.0099844.s001.pdf]

Supplemental UCSC genome browser session S1 'OccuPeak'

A supplemental UCSC genome browser session has been made accessible, enabling genome browser inspection of the results generated in this study. The session can be reached at: http://genome.ucsc.edu/cgi-bin/hgSession?hgS_doMainPage=1.

To load the session, go to the 'Restore Settings' field and enter:

1) user: AEF

2) session name: OccuPeak

If the load was successful, browsing can be started by clicking the Browser link in the 'Updated Session' field. In the 'Custom Tracks' menu you can select which tracks you want to visualize in the genome browser.
